# Identifying the determinants of health insurance coverage among Peruvian women of reproductive age: an assessment based on the national Peruvian demographic survey of 2017

**DOI:** 10.1186/s12939-020-01310-4

**Published:** 2020-11-03

**Authors:** Eduardo Ramos Rosas, Volker Winkler, Stephan Brenner, Manuela De Allegri

**Affiliations:** Heidelberg Institute of Global Health (HIGH), University Hospital and Medical Faculty, Heidelberg University, Im Neuenheimer Feld 130.3, 69120 Heidelberg, Germany

**Keywords:** Health insurance, Peru, Heath financing, SIS (Seguro integral de Salud), Women’s health

## Abstract

**Background:**

Like many other Latin America- and Caribbean countries, Peru has introduced a tax-financed health insurance scheme called “Sistema Integral de Salud (SIS)” to foster progress towards Universal Health Coverage. The scheme explicitly targets the poorest sections of the population. Our study explores levels of health insurance coverage and their determinants among Peruvian women following the introduction of SIS. We wish to determine the extent to which the introduction of SIS has effectively closed gaps in insurance coverage and for whom.

**Methods:**

Relying on the 2017 round of ENDES (Encuesta Nacional Demográfica y de Salud Familiar) survey, we analyzed data for 33,168 women aged 15–49. We used multinomial logistic regression to explore the association between health insurance coverage (defined as No Insurance, SIS, Standard Insurance) and women’s socio-demographic and economic characteristics.

**Results:**

Out of the 33,168 women, 25.3% did not have any insurance coverage, 45.5% were covered by SIS and 29.2% were covered by a Standard Insurance scheme. Women in the SIS group were found to have lower educational levels, live in rural areas and more likely to be poorer. Women in the Standard insurance group were found to be more educated, more likely to be “Spanish”, and to be wealthier. Most uninsured women appeared to belong to a middle class, not poor enough to be eligible for SIS, but also not eligible for standard insurance.

**Conclusions:**

Our study confirms that SIS has been effective in increasing coverage among vulnerable women, with coverage rates comparable with those observed among men. Nevertheless, on its own, it has proven to be insufficient to ensure universal coverage among women. Further reforms are needed to ensure that coverage is extended to all population groups.

## Background

One of the Sustainable Development Goals (SDG), specifically SDG3, adopted in 2015 by all United Nations Member States, is to “ensure healthy lives and promote well-being for all at all ages”. A specific target embedded in SDG3 is to achieve Universal Health Coverage (UHC) in all countries by 2030. The objective of UHC is to secure access to quality health services while ensuring financial risk protection in case of illness [1].

Over the last few decades, many countries in Latin America and the Caribbean (LAC) have been implementing health financing reforms aimed at fostering progress towards UHC. These reforms have largely aimed at increasing the number of people included in formal social health protection mechanisms (i.e. insurance schemes and tax-based health systems), mainly by expanding the range of health services covered by existing schemes [2]. Many LAC countries introduced health insurance schemes specifically targeting the poor and informal workers previously not covered by the existing social insurance schemes offered to formally employed workers [3]. In most settings, theses targeted schemes are often non-contributory for members with insurance contributions largely covered through public subsidies [2]. As a result, benefit packages and accessibility to provider networks are often more limited compared to the social insurance schemes offered to formally employed, thus leading to a some system segmentation, which in turn gives rise to important inequalities in terms of access and quality of care [4].

Following other LAC countries such as Colombia and Mexico [5], Peru implemented the “*Seguro Integral de Salud/SIS”* (translated as “Integrated Health Insurance”) in 2002 as a government-subsidized health insurance scheme aimed at providing coverage to uninsured citizens. SIS specifically targets the most vulnerable sections of the population, which are defined by the government to include those living in poverty or extreme poverty, as well as uninsured children and pregnant women [6]. SIS is only accessible to those residents not otherwise enrolled in any of the available insurance schemes. SIS enrolment process requires a valid Peruvian ID-document (for citizens) or Foreign Registration Card (for foreigners) to be presented to one of the SIS registration centers [7]. SIS is regulated by the Peruvian Ministry of Health (MINSA), which fully subsidizes insurance payments of the poorest, with partial subsidization for low income households of formal and informal workers. In case of partial subsidization, monthly insurance contributions to be paid range between 15 to 40 USD depending on household size [8]. The scheme provides access to an exclusive physician and hospital network [9] of approximately 430 hospitals and health posts distributed throughout the country. Since its creation, the percentage of the total population covered by SIS has increased from 17% in 2007 to around 47% in 2017, and is currently the largest health insurance scheme in Peru [10].

Alongside SIS, EsSalud, a standard contributory health insurance scheme regulated by the Ministry of Labour and Employment Promotion, provides mandatory coverage for all people employed in the formal sector [11] and represents the second most common insurance scheme (25%) [12], with around 380 healthcare centers (90 of them hospitals) distributed across the country. At least half of these hospitals are located in Lima [13]. Employers can also opt to provide additional coverage by enrolling in the *so-called EPS (Empresa Prestadora de Servicios*), the equivalent to a complementary private health insurance with a dedicated network of private providers, chosen by each EPS independently [14, 15] . Citizens serving in the marine, police or army (FFAA/Police) as well as their families have a special insurance and their own exclusive hospital and physician network [14]. Individuals can also purchase Private Health Insurance (PHI), as a complement to any other scheme (except the SIS) or as main insurance [16]. The percentage of people enrolled in EPS, FFAA/Police or PHI is around 5% [12]. All available insurance schemes in Peru must cover a list of essential services included in the *Plan Esencial de Aseguramiento Universal*, covering more than 1400 different conditions [17] [7].

The introduction of SIS has led to one of the most remarkable increases in coverage in Latin America, resulting in a vast expansion of primary healthcare coverage [3] by significantly reducing health-related out-of-pocket expenditures [18, 19]. However, little is understood about the persisting inequities in healthcare coverage in spite of the successful SIS implementation and its important contributions towards UHC in Peru. While in 2017 only 76% of all Peruvians enjoyed coverage through one of the above-mentioned schemes [20], little is known why about a fourth of the population (22% of women and 27% of men) [10] still continue to be without any formal social health protection.

In response to a recent call to generate evidence on women’s inclusion in social protection mechanisms as a result of recent health financing reforms [21, 22], we decided to focus our study exclusively on women of reproductive age, and aimed to fill this gap in knowledge by exploring the specific determinants of insurance coverage among them.. Limiting our sample exclusively to women of reproductive age rather than including women of all ages was dictated by data availability.

## Methods

### Study design and data

This cross-sectional study used data from the 2017 round of the *“Encuesta Nacional Demográfica y de Salud Familiar”* (ENDES), a nationally representative population-based survey designed for Peruvian women aged 15–49 years old. ENDES is carried out yearly by the Peruvian *“Instituto Nacional de Estadística e Informática”* (INEI) following the model and methodology of the Demographic and Health Survey (DHS) program [23]. The survey captures respondents’ socioeconomic status, demographics, household characteristics, insurance status, reproductive health, maternal health care, domestic violence, use of contraceptive methods, HIV prevalence, and vaccination status, as well as information about any under-five-year-old children and domestic partners [24].

Data used in this study was collected between March and December 2017, recollecting information from 35,190 Peruvian households with a total of 34,002 women surveyed, resulting in 33,168 completed questionnaires. This is also the total number of observations we included in our study. Completed interviews were registered in 97.5% of surveyed women [25]. None of the authors was directly involved in data collection and the team obtained fully-anonymized data directly from the INEI webpage [24]. For the analysis presented in this study, we used data from the respondents’ basic demographic and socio-economic sections and their insurance status. Data downloaded across sections were merged using the respondents’ unique identifiers.

### Measurements

Table 1 reports all variables used in our study, their measurement and the expected association with the outcome variable. “Health insurance status” served as the outcome variable and was defined as the specific health insurance coverage reported by each woman. It was coded as a categorical variable with three possible outcome values to reflect the insurance landscape of Peru: No Insurance, SIS, and Standard Insurance. Under “Standard Insurance”, we grouped women who reported to be covered by any formal sector scheme, such as EsSalud, Armed forces/Police Health Insurance (FFFAA/Police), EPS, but also PHI, either due to their own or their spouses’ formal employment. This categorization allowed us to explore the extent to which SIS effectively reached those women with no or informal employment targeted by the SIS.

**Table 1:** Summary of variables

Variable category	Variable	Categorization		
Outcome	Health insurance status	1 = No insurance 2 = SIS (“Integrated Health Insurance”) 3 = Standard Insurance (EsSalud, EPS, FFAA/Police, PHI)		
			Expected sign of association	
			SIS	Other insurance
Explanatory variables	Highest educational level attained	0 = Primary or no education 1 = Secondary 2 = Higher but no university 3 = Higher, university or Postgraduate	–	+
	Ethnicity	0 = Other 1 = Spanish	–	+
	Marital status	0 = Not currently married 1 = Currently married	+	+
	Place of residence	0 = Rural 1 = Urban	–	+
	Wealth index	0 = Poorest 1 = Poorer 2 = Middle 3 = Wealthier 4 = Wealthiest	–	+
	Currently working	0 = No 1 = Yes	–	+
	Births in last 5 years	0 = 0 births 1 = 1 or more births	+	+
	Total living children	0 = 0 children 1 = 1–2 children 2 = 3 or more children	+	–
	Household size	0 = 1–3 members 1 = 4–6 members 2 = 7 or more members	+	–
	Age	continuous	+	+

Selection of explanatory variables was informed by prior studies that identified their relationship to health insurance coverage [26–32]. Most explanatory variables are self-explanatory. “Ethnicity” represents a pre-generated self-identification variable in the INEI defined by respondents’ reported language, birthplace, cultural traditions and information pertaining to their ancestors and relatives [33]. We further condensed this variable into two categories: “Spanish” as the most commonly reported ethnicity, and “Other” including all other reported ethnicities (i.e. Quechua, Aymara, other indigenous or foreign ethnicities).

“Wealth index” at household level was also pre-generated by the INEI and based on a composite measure that reflects households’ living conditions and ownership of a variety of assets, following the model established by the DHS [34, 35] . Based on this index, we classified women in five quintiles from “Poorest” to “Wealthiest”.

### Analytical approach

First, we performed descriptive analyses to explore the distribution of both outcome and explanatory variables (Table 2). Second, we performed bivariate analyses using chi-squared test of independence for categorical and ANOVA for continuous variables to determine those explanatory variables to be included in our model (Table 3). Across all analyses, individual information was adjusted to ensure population representativeness using the weights provided by the INEI [24]. Third, we performed a multinomial logistic regression (MNLR) to identify determinants of health insurance coverage using “No Insurance” as base category by comparing it to “SIS” and to “Standard Insurance”, respectively. The model included the time-invariant variable “Region” to fix effects due to variances attributable to regional-level characteristics.

**Table 2:** Sample characteristics (*N* = 33,168)

***Outcome variable***	N	%
**Health insurance status**		
No insurance	8391	25.3
SIS	15,087	45.5
Standard Insurance (EsSalud, FFAA/Police, EPS, Private)	9690	29.2
***Explanatory variables***		
**Highest educational level attained**		
Primary or no education	5969	17.9
Secondary	15,140	45.7
Higher but no university	6068	15.3
Higher, university or Postgraduate	5991	18.1
**Ethnicity**		
Other	2117	6.4
Spanish	31,051	93.6
**Marital status**		
Not currently married	14,395	43.4
Currently married	18,773	56.6
**Place of residence**		
Rural	6432	19.4
Urban	26,736	80.6
**Wealth index**		
Poorest	5535	17.0
Poorer	6842	20.6
Middle	7127	21.5
Wealthier	6943	20.9
Wealthiest	6720	20.3
**Currently working**		
No	12,128	36.6
Yes	21,040	63.4
**Births in last 5 years**		
0	23,418	70.6
1+	9750	29.4
**Total living children**		
0	10,582	31.9
1–2	14,455	43.6
3+	8131	24.5
**Household size**		
1–3	9402	28.4
4–6	19,088	57.6
7+	4678	14.0
**Age**	**Mean**	**SD**
15–49 (continuous)	31.3	9.8

**Table 3:** Bivariate analysis

Variable	Health insurance status			
Highest educational level attained	No Insurance (% column)	SIS (% column)	Standard Insurance (% column)	Chi-square ***p***-value
Primary or no education	13.0	29.2	4.8	< 0.001
Secondary	47.9	51.6	34.4	
Higher but no university	19.6	12.5	26.2	
Higher, university or Postgraduate	19.4	6.7	34.6	
**Ethnicity**				
Other	3.8	11.1	1.3	< 0.001
Spanish	96.2	88.9	98.7	
**Marital status**				
Not currently married	54.2	37.9	45.6	< 0.001
Currently married	45.8	62.1	57.4	
**Place of residence**				
Rural	11.3	33.4	4.7	< 0.001
Urban	88.7	66.6	95.3	
**Wealth index**				
Poorest	8.3	31.0	1.7	< 0.001
Poorer	18.8	28.4	10.0	
Middle	23.7	21.5	19.7	
Wealthier	26.1	12.8	29.0	
Wealthiest	23.1	6.3	39.6	
**Currently working**				
No	36.4	42.4	27.7	< 0.001
Yes	63.6	57.7	72.3	
**Births in last 5 years**				
0	79.7	63.4	73.9	< 0.001
1+	20.3	36.6	26.1	
**Total living children**				
0	41.1	26.0	33.1	< 0.001
1–2	37.4	43.8	27.7	
3+	24.5	30.2	18.2	
**Household size**				
1–3	30.5	26.8	28.9	< 0.001
4–6	55.7	57.1	59.9	
7+	13.8	16.1	11.2	
**Age**	**No insurance** **Mean (SD)**	**SIS** **Mean (SD)**	**Standard Insurance** **Mean (SD)**	**Anova (*p*-value)**
15–49 (continuous)	30.61 (9.97)	30.41 (9.85)	33.11 (9.44)	< 0.001

The analytical model can be expressed as [36]:

$$ \Pr\ \left({Y}_i=M\right)=\frac{\mathit{\exp}\ \left({\beta}_{Mi}{x}_i\right)}{\sum \limits_1^M\mathit{\exp}\left({\beta}_{1i}{x}_i\right)}, for\ M=1,2\  or\ 3 $$

Here “*M* = 1” refers to “No Insurance”, “*M* = 2” to “SIS” and “*M* = 3” to “Standard Insurance”. We selected “No Insurance” as a base category to ease interpretations of the findings by focusing on conceptually relevant comparisons.

Results are presented in terms of relative risk ratios (RRR), 95% confidence intervals, and relevant *p*-values. To test if the model meets the assumption of independence of irrelevant alternatives (IIA), meaning that the addition or deletion of variables should not affect the results showed in the regression, we performed a Small-Hsiao test [37], which confirmed the IIA assumption was not violated. All statistical analysis was performed using STATA 15.1.

## Results

### Descriptive statistics

Table 2 summarizes sample characteristics. Out of a total of 33,168 women included in our sample, 25.3% reported no insurance coverage, 45.5% were affiliated to SIS and 29.2% had Standard Insurance. Average age was 31 years, with a SD of 9.8. Nearly 80% of women surveyed reported a completed secondary education or higher. Most women were identified as “Spanish” (93.6%), were married (56.6%), urban residents (80.6%) and were working in the week prior to the survey (63.4%). Around 30% of women had given birth to one or more children in the 5 years prior to the survey.

Findings from the bivariate analysis (Table 3) indicated the existence of significant associations between all explanatory variables and the outcome variable “health insurance status”. The average age of women in the SIS group was 30.4, with a SD of 9.9. They were found to have lower educational levels (around 80% with secondary as the highest educational level attained), belonged to other ethnicities (11.1%), lived in rural areas (33.4%), were poorer and were not working (42.4%).

On the other hand, women in the “Standard Insurance” group had an average age of 33.1 (SD = 9.4) and were found to be more educated (around 50% with higher education than secondary). Most of them identified as “Spanish” (98.7%), lived in urban settings (95.3%), were wealthier and were working when the data was collected. (72.3%).

More than 75% of the uninsured women reported at least “Secondary” as the highest educational level attained, were identified as “Spanish”, belonged to a wealth index group higher than “poorer” and reported to live in urban settings. The proportion of marriage, living children and births in the 5 years prior to the survey was reported to be lower in this group than in the other groups.

Results of the MNLR (Table 4) confirmed that compared to women with no insurance, women with “SIS” coverage were younger (RRR = 0.99), less likely to be identified as Spanish (RRR = 0.78), less likely to have completed secondary- (RRR = 0.84), higher- (RRR = 0.65) or university education (RRR = 0.51), less likely to reside in urban settings (RRR = 0.85), less likely to belong to wealthier groups (RRR = 0.28 for “Wealthier” and RRR = 0.18 for “Wealthiest”), and less likely to have been working (RRR = 0.86). Having three or more living children and belonging to a household with seven or more members also increased the likelihood to belong to the SIS group (RRR = 1.23 and 1.20 respectively).

**Table 4:** Results from the multinomial logistic model using “No Insurance” as base outcome

	SIS	Standard Insurance
**Highest educational level attained**		
Primary (Ref.)	1.00	1.00
Secondary	0.84 (0.76–0.92) ***	1.76 (1.55–2.01) ***
Higher, but no university	0.65 (0.58–0.73) ***	2.50 (2.17–2.87) ***
Higher, university or Postgraduate	0.51 (0.45–0.58) ***	3.65 (3.16–4.22) ***
**Ethnicity**		
Other: Quechua, Aymara, etc. (Ref.)	1.00	1.00
Spanish	0.78 (0.67–0.90) **	1.56 (1.24–1.98) ***
**Marital status**		
Not currently married (Ref.)	1.00	1.00
Currently married	1.20 (1.11–1.29) ***	1.61 (1.49–1.74) ***
**Place of residence**		
Rural (Ref.)	1.00	1.00
Urban	0.85 (0.76–0.96) ***	0.87 (0.75–1.02) *
**Total births in last 5 years**		
0 (Ref.)	1.00	1.00
1+	1.54 (1.42–1.67) ***	1.29 (1.18–1.41) ***
**Total living children**		
0 (Ref.)	1.00	1.00
1–2	1.39 (1.25–1.54) ***	1.03 (0.93–1.14)
3+	1.23 (1.07–1.40) ***	0.80 (0.70–0.91) ***
**Wealth index**		
Poorest (Ref.)	1.00	1.00
Poorer	0.61 (0.54–0.69) ***	2.15 (1.74–2.66) ***
Middle	0.46 (0.40–0.52) ***	3.05 (2.44–3.80) ***
Wealthier	0.28 (0.24–0.32) ***	3.53 (2.82–4.42) ***
Wealthiest	0.18 (0.15–0.21) ***	4.74 (3.77–5.97) ***
**Currently working**		
No (Ref.)	1.00	1.00
Yes	0.86 (0.81–0.92) **	1.24 (1.15–1.33) ***
**Household size**		
1–3 (Ref.)	1.00	1.00
4–6	1.13 (1.06–1.21) ***	1.15 (1.07–1.23) **
7+	1.20 (1.09–1.32) ***	0.96 (0.86–1.07) *
**Age (cont.)**	0.99 (0.98–0.99) ***	1.03 (1.02–1.03) ***
*Constant*	6.36 (5.15–7.86) ***	0.04 (0.03–0.05) ***
*Pseudo R2*	0.18	
*Total observations*	33,168	

Note: Relative Risk Ratio and 95% confidence intervals are shown. “No Insurance” was used as base outcome. Significance level:

***: *p* < 0.01; **: *p* < 0.05; *: *p* < 0.10

Meanwhile, women with “Standard Insurance” coverage were more likely to be older (RRR = 1.03 for each increasing year of age), more likely to be better educated (RRR = 2.50 for higher-but-not university and RRR = 3.65 for university or postgraduate), more likely to be “Spanish” (RRR = 1.56), more likely to be wealthier (RRR = 3.53 and 4.74), and to have been working (RRR = 1.24). Contrary to our expectations, the MNLR reported urban resident women to be less likely to have “Standard Insurance” (RRR = 0.87).

## Discussion

This study makes an important contribution to the existing literature as one of few studies addressing factors associated with health insurance coverage in Peru. While prior studies have primarily focused on examining effects of different insurance schemes on health coverage and service access [38], as well as financial protection [18, 19], no explicit examination of determinants of insurance coverage in Peru has been reported so far. With its narrow focus on women, our study also contributes to the rather limited literature on gender aspects in health financing, such as the identification of coverage gaps specifically faced by women [22].

Our study confirmed almost all the expected associations indicated in Table 1, except for “Age”, where every increasing year of age made it more likely that the individual would have “Standard Insurance” and decreased the likelihood of them having SIS coverage.

Our findings reveal that in spite of the efforts made by the government over the last few years, a relatively large proportion of women of reproductive age (about 25%) still lacks insurance coverage and as such have limited financial risk protection in case of illness. This value is aligned with official population-based estimates, suggesting that following the introduction of SIS in the early 2000s, the proportion of uninsured people decreased from 57.7% in 2007 to 24.5% in 2017 [12], and in the case of women to around 22% in 2017 [39]. The results from our study are consistent with national estimates, which reported that the proportion of uninsured women was lower (22.1%) compared with men (27.2%) [10]. Based on these estimates, we further conclude that women likely did not face stronger barriers to enrolling under SIS compared to men.

Our findings are worrisome since compared to other LAC countries, such as Colombia, Brazil or Chile, where insurance coverage has reached 90% [40, 41], Peru still lags behind when it comes to securing social health protection through publicly funded insurance schemes. However, findings from these countries is not stratified by population groups and therefore may hide inequities in respect to gender and therefore not fully comparable to our current findings.

This coverage gap may appear surprising given that SIS was launched with the specific intention of fostering progress towards UHC by increasing insurance coverage. However, since it launch the SIS has remained a targeted scheme ensuring coverage of vulnerable populations working in the informal sector in general, not women per se [6]. Highlighting the existence of a positive association between SIS coverage and lower socio-economic status, lower education, rural settings and no current employment, our findings suggest that SIS was nevertheless largely successful in reaching a considerable portion of the Peruvian population it intended to reach. Yet, and contrary to our expectations, women living in urban settings were also less likely to belong to the “Standard Insurance” group. While this might be due to increases in low-income earners across cities, further research would be needed to fully explain this finding.

Still surprising, uninsured women were neither necessarily the poorest and less educated, nor the highest educated and richest. Rather, they seemed to represented a middle-class stratum of urban women characterized by being single, of Spanish ethnicity, with few or no children compared with insured women. This finding might directly point at problems related to fragmented health financing structures. While Peru, similar to other LAC countries, kept some level of fragmentation in its financing and organization of health systems by introducing a separate tax-financed insurance scheme targeting the poor [2, 42], the co-existence of multiple pools, each targeting a specific segment of the population, inevitably leaves the most vulnerable groups more exposed to the risk associated with falling ill [43], resulting in inequities and inefficiencies in health coverage [44, 45].

One of the remaining challenges for the Peruvian Government in the coming years is to more specifically target these various population groups to overcome persisting inequities in the country [46]. So far, the government has introduced an additional insurance package within the SIS that also allows workers from the informal sector, who don’t necessarily live in extreme poverty, to enroll by paying small monthly contributions that enable access to the SIS physician and hospital network nationwide. However, related research indicates that in order to reduce inequities there needs to be a stronger focus on increasing the amount of pooled finances in the current context of multiple co-existing insurance pools [2, 47].

### Methodological considerations

The key strengths of this study lie in its large sample size and the resulting analytical robustness. Nevertheless, we must acknowledge several weaknesses. First, as we relied on secondary data, our sample is limited to women of reproductive age (15–49 years old), thus not allowing any insight on insurance coverage of older women in the country. We cannot exclude the possibility that different, possibly lower coverage rates might pertain to older women, possibly due to gaps in their knowledge of their entitlements. Second, given the reliance on secondary data, we were limited to variables available in the original survey. For instance, we could not look at the role distance to public health facilities might have played in determining insurance coverage in Peru. Similarly, we were unable to include any information on household heads and the extent to which health-related decision making at the household level hence might have determined women’s insurance status.

## Conclusions

In line with existing literature, our study confirms that the introduction of government-subsidized schemes has surely contributed to increasing financial protection for the most vulnerable in Peru. However, targeting specifically the poorest and most vulnerable has left a significant group of the women uninsured, generating inequities in health access. SIS has fulfilled its goal as a targeted scheme and its creation has surely fostered progress toward UHC, but on its own, it has proven to be insufficient to reach universal coverage. Further reforms are needed to offer coverage to currently uninsured women, either through expansion of existing schemes or by reducing fragmentation in resource generation and integrating multiple population sectors into a single scheme.

## Data Availability

The datasets analyzed during the current study are available in the INEI repository and are freely accessible under http://webinei.inei.gob.pe/anda_inei/index.php/catalog/649/datafile/F4/V131

## Abbreviations

SDG: Sustainable development goals
UHC: Universal health coverage
LAC: Latin America and the Caribbean
SIS: “Seguro Integral de Salud”
MINSA: Peruvian ministry of health
EPS: “Empresa Prestadora de Servicios”
FFAA/Police: Marine, police or army
ENDES: “Encuesta Nacional Demográfica y de Salud Familiar”
INEI: “Instituto Nacional de Estadística e Informática”
DHS: Demographic and health survey
PHI: Private health insurance
MNLR: Multinomial logistic regression
IIA: Independence of irrelevant alternatives
RRR: Relative-risk ratio
